# Vocal Music Listening Enhances Poststroke Language Network Reorganization

**DOI:** 10.1523/ENEURO.0158-21.2021

**Published:** 2021-07-06

**Authors:** Aleksi J. Sihvonen, Pablo Ripollés, Vera Leo, Jani Saunavaara, Riitta Parkkola, Antoni Rodríguez-Fornells, Seppo Soinila, Teppo Särkämö

**Affiliations:** 1 Cognitive Brain Research Unit, Department of Psychology and Logopedics, Faculty of Medicine, University of Helsinki, FI-00014 Helsinki, Finland; 2 Centre for Clinical Research, The University of Queensland, Herston 4029, Queensland, Australia; 3 Department of Psychology, New York University, New York, New York 10003; 4 Music and Audio Research Laboratory, New York University, Brooklyn, New York 11201; 5 Center for Language, Music and Emotion, New York University, New York, New York 10014; 6 Department of Medical Physics, Turku University Hospital, 20521 Turku, Finland; 7 Department of Radiology, Turku University Hospital and University of Turku, FI-20520 Turku, Finland; 8 Cognition and Brain Plasticity Group, Bellvitge Biomedical Research Institute, L’Hospitalet de Llobregat, 08908 Barcelona, Spain; 9 Department of Cognition, Development and Education Psychology, University of Barcelona, 08035 Barcelona, Spain; 10 Institució Catalana de Recerca i Estudis Avançats, 08037 Barcelona, Spain; 11 Neurocenter, Turku University Hospital and Division of Clinical Neurosciences, University of Turku, 20521 Turku, Finland

**Keywords:** aphasia, DTI, fMRI, language, music, recovery

## Abstract

Listening to vocal music has been recently shown to improve language recovery in stroke survivors. The neuroplasticity mechanisms supporting this effect are, however, still unknown. Using data from a three-arm, single-blind, randomized controlled trial including acute stroke patients (*N* = 38) and a 3 month follow-up, we set out to compare the neuroplasticity effects of daily listening to self-selected vocal music, instrumental music, and audiobooks on both brain activity and structural connectivity of the language network. Using deterministic tractography, we show that the 3 month intervention induced an enhancement of the microstructural properties of the left frontal aslant tract (FAT) for the vocal music group compared with the audiobook group. Importantly, this increase in the strength of the structural connectivity of the left FAT correlated with improved language skills. Analyses of stimulus-specific activation changes showed that the vocal music group exhibited increased activations in the frontal termination points of the left FAT during vocal music listening compared with the audiobook group from acute to 3 month poststroke stage. The increased activity correlated with the structural neuroplasticity changes in the left FAT. These results suggest that the beneficial effects of vocal music listening on poststroke language recovery are underpinned by structural neuroplasticity changes within the language network and extend our understanding of music-based interventions in stroke rehabilitation.

## Significance Statement

Poststroke language deficits have a devastating effect on patients and their families. Current treatments yield highly variable outcomes, and the evidence for their long-term effects is limited. Patients often receive insufficient treatment that is predominantly given outside the optimal time window for brain plasticity. Poststroke vocal music listening improves language outcome, which is underpinned by neuroplasticity changes within the language network. Vocal music listening provides a complementary rehabilitation strategy that could be safely implemented in the early stages of stroke rehabilitation and seems to specifically target language symptoms and recovering language network.

## Introduction

Rapid aging of the population leads to a massive growth in the prevalence of stroke (Feigin et al., 2014), which incurs enormous socioeconomical challenges (Olesen et al., 2012). Poststroke aphasia—an impairment of speech production and/or comprehension—occurs in up to 40% of stroke patients (Pedersen et al., 2004) and has a devastating impact on the individual, decreasing quality of life more than any other stroke-induced impairment (Lam and Wodchis, 2010).

Language functions are underpinned by a left-lateralized network comprising frontal, temporal, and parietal brain regions and the white matter pathways interconnecting them (Hickok and Poeppel, 2007; Saur et al., 2008; Rauschecker and Scott, 2009; Alyahya et al., 2020; Hula et al., 2020). In poststroke language impairments, the language network is disrupted because of hypoperfusion and consequent brain tissue damage (Fox, 2018), and recovery relies on the ability of the spared neurons to remodel the injured network (Kiran et al., 2019). Aphasia treatments aim to achieve functional gains by promoting neuroplasticity processes within the language network (Cramer, 2008, 2018). Better aphasia outcomes have been associated with functional neuroplasticity changes within the language network, mainly in the left hemisphere, during both spontaneous recovery (Saur et al., 2006) and after treatments (Fridriksson, 2010; Fridriksson et al., 2012; Van Hees et al., 2014a; Hartwigsen and Saur, 2019). Studies evaluating treatment-related structural connectivity changes in aphasia are sparse, but have linked better outcomes with plasticity changes in the left-hemispheric white matter tracts (Van Hees et al., 2014b).

Current treatments have, however, shown highly variable outcomes and the evidence for their long-term effects is scarce (Brady et al., 2016). It is vital to pursue new rehabilitation methods that are inexpensive and both independent of and complementary to the traditional rehabilitation strategies. In this vein, music-based interventions have emerged as promising rehabilitation strategies in many neurologic diseases, including stroke (Winstein et al., 2016; Sihvonen et al., 2017c). In neurologic rehabilitation, music provides a multidomain stimulus that increases activity-dependent neuroplasticity in the brain and provides a fertile environment for recovery (Murphy and Corbett, 2009; Särkämö and Soto, 2012). In stroke patients, daily music listening during the subacute poststroke stage has been found beneficial for improving cognitive and emotional recovery (Särkämö et al., 2008; Baylan et al., 2020) and increasing gray matter volume in frontolimbic regions (Särkämö et al., 2014) compared with standard care. Recently, using data pooled together from two randomized controlled trials (RCTs), we showed that the vocal (sung) component of music is crucial for its rehabilitative efficacy: compared with instrumental music and audiobooks, vocal music listening improved language recovery and verbal memory, especially in patients with aphasia, and was coupled with increased gray matter volume in temporal regions and enhanced functional connectivity of the default mode network (Sihvonen et al., 2020).

While vocal music listening promotes poststroke language recovery, little is known about the specific language-related neural mechanisms supporting this effect. An interesting hypothesis is that vocal music listening induces neuroplasticity effects on the language network, especially in the regions linked to connected speech after stroke (e.g., the left frontal regions and their underlying white matter tracts; Alyahya et al., 2020). Evaluating the possible neurobiological mediators of recovery and treatment effects is of great importance for improving our understanding of aphasia rehabilitation, and for optimizing current and future approaches (Copland, 2020).

The present study sought to unveil the neuroplasticity effects of vocal music in both the brain function and the structural connectivity of the language network. To do so, we assessed longitudinally a subsample of 38 stroke patients from our previous music intervention study (Sihvonen et al., 2020) using diffusion-weighted imaging (DWI) and task-related functional MRI (fMRI). We hypothesized that poststroke vocal music listening induces neuroplasticity changes in the language network that, in turn, underpin the enhanced recovery of language skills (Sihvonen et al., 2020).

## Materials and Methods

### Subjects and study design

Fifty stroke patients were recruited from 2013 to 2016 from the Turku University Hospital for a three-arm RCT (ClinicalTrials.gov: trial NCT01749709). Inclusion criteria were acute unilateral stroke; right handedness; age <80 years; capability to communicate in Finnish; residence in Southwest Finland; ability to cooperate; and normal hearing. Patients with prior neurologic or psychiatric disease or substance abuse were not included. The study was approved by the Ethics Committee of the Hospital District of Southwest Finland and performed in conformance with the Declaration of Helsinki. All patients gave informed consent, and received standard stroke treatment and rehabilitation. Baseline MRI scans and behavioral assessments were performed <3 weeks poststroke (mean, 12 d; SD, 5.5). Patients were then randomly allocated to vocal music group (VMG; *N* = 17), instrumental music group (IMG; *N* = 17), and audiobook group (ABG; *N* = 16). The randomization was stratified for lesion laterality (left/right) and performed as block randomization (10 blocks of three consecutive patients for left and right lesions), with the order within the blocks being drawn by a random number generator. The randomization list was generated by a laboratory engineer not involved in the data collection and the persons performing the patient recruitment had no access to it (allocation concealment). During follow-up, six patients were excluded because of refusal to participate at follow-up, and six patients because of incomplete MRI data. Thirty-eight of the remaining patients (15 female and 23 male; mean age, 56.1 years; SD, 13.4) completed the intervention and 3 month postintervention MRI and behavioral assessments, and were included in statistical analyses (VMG, *N* = 12; IMG, *N* = 15; ABG, *N* = 11; Table 1). The groups did not differ between clinical variables (Table 1) such as stroke type (*p* = 0.398), lesion laterality (*p* = 0.676), or lesion volume (*p* = 0.712), nor did the study groups differ between the National Institutes of Health Stroke Scale scores at the acute stage (*F*_(22,38)_ = 0.872, *p* = 0.627; Wilks’ lambda = 0.442; individual categories. *p* = 0.153–0.994). Patients with both ischemic and hemorrhagic strokes were included to reflect the real-world clinical population undergoing rehabilitation as well as to increase the generalization of the effects of this intervention. In clinical populations, the prevalence of poststroke cognitive impairments has not been shown to differ between ischemic and hemorrhagic strokes (Lo et al., 2019).

**Table 1:** Baseline demographic and clinical characteristics of the patients

	Vocal music group (*N* = 12)	Instrumental music group (*N* = 15)	Audiobook group (*N* = 11)	*p* Value
Demographic				
Sex (male/female)	5/7	11/4	7/4	0.239 (χ^2^)
Age (years)	54.1 (16.9)	53.6 (10.3)	62.0 (12.0)	0.218 (*F*)
Education (years)	14.7 (3.6)	13.8 (3.9)	12.5 (4.4)	0.450 (*F*)
Music background (prestroke)				
Formal music training^*a*^	0.6 (1.5)	0.0 (0.0)	0.9 (1.9)	0.218 (H)
Instrument playing^*a*^	1.8 (2.4)	1.2 (1.9)	1.8 (2.4)	0.762 (H)
Music listening prior to stroke^*a*^	4.5 (1.0)	4.9 (0.3)	4.1 (1.6)	0.265 (H)
Clinical				
Stroke type (infarct/haemorrhage)	10/2	9/6	7/4	0.398 (χ^2^)
Verbal fluency^*b*^	8.4 (5.4)	9.5 (5.3)	8.3 (3.3)	0.715 (H)
Naming^*c*^	18.3 (1.8)	17.5 (2.0)	17.4 (1.7)	0.444 (H)
Auditory comprehension^*d*^	27.5 (5.5)	27.7 (3.5)	24.6 (5.1)	0.112 (H)
Amusia overall^*e*^ (no/yes)	5/7	9/6	2/9	0.103 (χ^2^)
Amusia scale^*f*^ (no/yes)	5/7	10/5	5/6	0.370 (χ^2^)
Amusia rhythm^*g*^ (no/yes)	3/9	7/8	0/11	**0.028 (χ^2^)**
Lesion laterality (left/right)	6/6	7/8	7/4	0.676 (χ^2^)
Lesion volume (cm^3^)	49.0 (54.1)	66.0 (53.8)	55.6 (55.7)	0.712 (*F*)

Data are the mean (SD), unless otherwise stated. Significant group differences are shown in bold. *F*, One-way ANOVA; H, Kruskal–Wallis test; χ², χ^2^ test.

a Likert scale 0–5 (0, never; 1, rarely; 2, once a month; 3, once a week; 4, two to three times a week; 5, daily).

b Classification based on Verbal Fluency Test.

c Classification based on shortened Boston Naming test.

d Classification based on shortened Token Test.

e Classification based on the MBEA Scale and Rhythm subtest average score (<75% cutoff).

f Classification based on the MBEA Scale subtest score (<73% cutoff).

g Classification based on the MBEA Rhythm subtest score (<77% cutoff).

### Intervention

After baseline assessments, each patient was contacted by a professional music therapist who informed them of their group allocation and interviewed them about prestroke leisure activities, including music listening and reading. Other researchers were blinded to the group allocation of the patients. The therapist provided the patients with a portable MP3 player, headphones, and a collection of listening material individually selected to match the music or literature preferences of the patient as closely as possible. The listening material was vocal music with sung lyrics in VMG, instrumental music (with no sung lyrics) in IMG, and narrated audiobooks (with no music) in ABG. All material was in a language that the patients understood best (mostly Finnish or English). The patients were trained in using the players, instructed to listen to the allocated material by themselves daily (minimum 1 h/d) for the following 2 months in the hospital or at home, and asked to keep a listening diary. During the 2 month intervention period, the music therapist kept regular contact with the patients to encourage listening, provide more material, and help with the equipment if needed.

### MRI data acquisition

Patients were scanned on a 3 T Siemens Magnetom Verio scanner with a standard 12-channel head matrix coil at the Department of Radiology of Turku University Hospital. The MRI protocol comprised high-resolution T1-weighted anatomic images, DWI data (TR = 11,700 ms; TE = 88 ms; acquisition matrix = 112 × 112; 66 axial slices; voxel size = 2.0 × 2.0 × 2.0 mm^3^) with one non-diffusion-weighted volume and 64 diffusion-weighted volumes (b = 1000 s/mm^2^), and task-fMRI using a single-shot T2*-weighted gradient-echo EPI sequence (280 functional volumes; 32 slices; slice thickness = 3.5 mm; TR = 2010 ms; TE = 30 ms; flip angle = 80°; voxel size = 2.8 × 2.8 × 3.5 mm^3^).

During a block design task-fMRI, the patients were presented with 15 s excerpts of well known Finnish songs with (1) sung lyrics (vocal, 6 blocks) and (2) without sung lyrics (instrumental, 6 blocks), (3) well known Finnish poems (speech, 6 blocks), and (4) no auditory stimuli (rest, 18 blocks) through MR-compatible headphones using Presentation software (version 16.3, Neurobehavioral Systems). The order of the auditory blocks was randomized across subjects and time, and the rest blocks were presented in between the auditory blocks. Intervention listening material was not used in the task-fMRI excerpts.

### MRI data preprocessing

MRI data were preprocessed using Statistical Parametric Mapping software [SPM8, Wellcome Department of Cognitive Neurology, UCL (www.fil.ion.ucl.ac.uk/spm/)] under MATLAB version 8.4.0. The fMRI images were initially realigned, and a mean image of the whole task-fMRI run was created. Individual images were reoriented according to the anterior commissure. Cost function masking was applied to achieve optimal normalization of the lesioned brain tissue, with no postregistration lesion shrinkage or out-of-brain distortion (Brett et al., 2001; Andersen et al., 2010; Ripollés et al., 2012). Cost function masking was performed by manually depicting the stroke lesions slice by slice to the individual T1 images using MRIcron software package (http://people.cas.sc.edu/rorden/mricron/index.html; Rorden and Brett, 2000). All lesion tracing was conducted by one person (author A.J.S.) experienced in this matter (Sihvonen et al., 2016, 2017a). Task-fMRI data were normalized to Montreal Neurological Institute space using Unified Segmentation (Ashburner and Friston, 2005) and resampled into isotropic 2 × 2× 2 mm^3^ voxel size. Finally, the preprocessed task-fMRI data were smoothed using an isotropic spatial filter (FWHM = 8 mm).

The statistical evaluation of the task-fMRI data was based on a least-squares estimation using the general linear model at both time points (acute/3 month). At the individual level, the different task conditions (vocal/instrumental/speech) were modeled with a box-car regressor waveform convolved with a canonical hemodynamic response function. Data were high-pass filtered to a maximum of 1/128 Hz, and serial autocorrelations were estimated using an autoregressive model (AR[1] model). In addition, confounding factors from head movement were included in the model. A block-related design matrix was created including the conditions of interest (Vocal/Instrumental/Speech). After model estimation, main effects for each condition against rest were calculated (e.g., Vocal > Rest).

### DTI data preprocessing

The processing of DWI data started by correcting eddy current distortions and head motion using the FMRIB Software Library [FSL version 5.0.8, University of Oxford (www.fmrib.ox.ac.uk/fsl); Smith et al., 2004; Jenkinson et al., 2012]. Next, the gradient matrix was rotated using FSL fdt rotate bvecs to provide a more accurate estimate of diffusion tensor orientations (Leemans and Jones, 2009). Following this, brain extraction was performed using the Brain Extraction Tool (Smith, 2002). Then, the diffusion tensors were reconstructed using the linear least-squares algorithm included in Diffusion Toolkit version 0.6.2.2 (Ruopeng Wang, Van J. Wedeen (trackvis.org/dtk), Martinos Center for Biomedical Imaging, Massachusetts General Hospital, Charlestown, MA).

Dissections of individual white matter tracts were performed using TrackVis (version 0.6.0.1, Build 2015.04.07) following commonly used published guidelines for the number and positioning of the regions of interest (Catani et al., 2002; Sihvonen et al., 2017b). All analyses were performed by one person (author A.J.S.) experienced in virtual dissections (Sihvonen et al., 2017b). Deterministic tractography analysis focused on three white matter tracts integral to the language network and language skills (Catani et al., 2005, 2012; Dick and Tremblay, 2012; Dick et al., 2019; Alyahya et al., 2020): the left arcuate fasciculus (AF; long segment), the inferior fronto-occipital fasciculus (IFOF), and the left frontal aslant tract (FAT). After dissections, fractional anisotropy (FA) values of each tract, representing white matter integrity, were collected using MATLAB toolbox, “along-tract statistics” (Colby et al., 2012) and imported into IBM SPSS Statistics 27. Lower FA values in left-hemispheric tracts have been associated with greater aphasia severity (Rosso et al., 2015). Deterministic tractography dissections and placement of regions of interest have been described in detail previously (Sihvonen et al., 2017b).

### Language assessment

Language assessment was performed in both time points (acute, 3 months) using the standard Verbal Fluency Test (Lezak et al., 2012); the shortened Token Test (De Renzi and Faglioni, 1978); and the shortened Boston Naming test (a 20-item version including every third of the original 60 line drawings with a maximum score of 20; Morris et al., 1989; Laine et al., 1993), blinded to the group allocation of the patient. In our previous study (Sihvonen et al., 2020), the individual tests correlated significantly with each other (acute stage in all: *r* = 0.46–0.84, *p* < 0.001) and a summary score was calculated by adding up the raw test scores used in the analyses. To follow our previous study, and to maintain continuity and uniformity, as well as to conform the smaller sample size, the summary score was used in the current analyses.

### Statistical analyses

In deterministic tractography analysis, multivariate ANOVA with change (3 month minus acute) in FA values of the left AF, IFOF, and FAT as dependent variables and Group as a factor was performed. Total brain volume (TBV) and cross-listening (i.e., listening to material not part of the protocol) were included as covariates in the analysis (Sihvonen et al., 2020). In addition, the groups showed significant differences in the prevalence of rhythm amusia (*p* = 0.028; Table 1), and therefore the Montreal Battery of Evaluation of Amusia (MBEA) Rhythm Subtest score (Peretz et al., 2003) was also included as a covariate in the analysis. Results were corrected for *post hoc* multiple comparisons using the Bonferroni adjustment.

Statistical analyses of the preprocessed task-fMRI data were conducted using SPM8. To evaluate longitudinal changes, three flexible factorial ANOVAs with Time (Acute/3 month) and Group (VMG/IMG/ABG) as factors were performed using the Vocal > Rest, Instrumental > Rest, and Speech > Rest conditions. To focus analyses on the language network, analyses were constricted to the left hemisphere using an explicit mask. All task-fMRI result (spmT) maps were thresholded at an uncorrected *p* < 0.001 at the voxel level, and standard SPM familywise error cluster-level correction based on random field theory with a *p* value <0.05 was used (Eklund et al., 2016). Because of three conditions, α-level was set to *p* < 0.017, and only clusters surviving this threshold are reported. Similar to tractography, TBV, cross-listening, and MBEA Rhythm Subtest score were included as covariates in the task-fMRI analysis (Table 1).

Correlation analyses (Spearman, two‐tailed) were performed between the significant tractography and fMRI changes (3 month > Acute) and the changes in language skills.

### Data availability

The data that support the findings of this study are available from the corresponding author, A.J.S., on reasonable request.

## Results

### Structural neuroplasticity

In the multivariate ANOVA, there was a statistically significant difference in the longitudinal change (3 month > Acute) in the microstructural properties of the left-hemispheric tracts between the groups (*F*_(6,60)_ = 2.859; *p* = 0.016; Wilks’ lambda = 0.605, partial η^2^ = 0.222). Therefore, three separate univariate ANOVAs (i.e., one for each tract) were performed, and α-level was set to *p* < 0.017.

**Figure 1.**
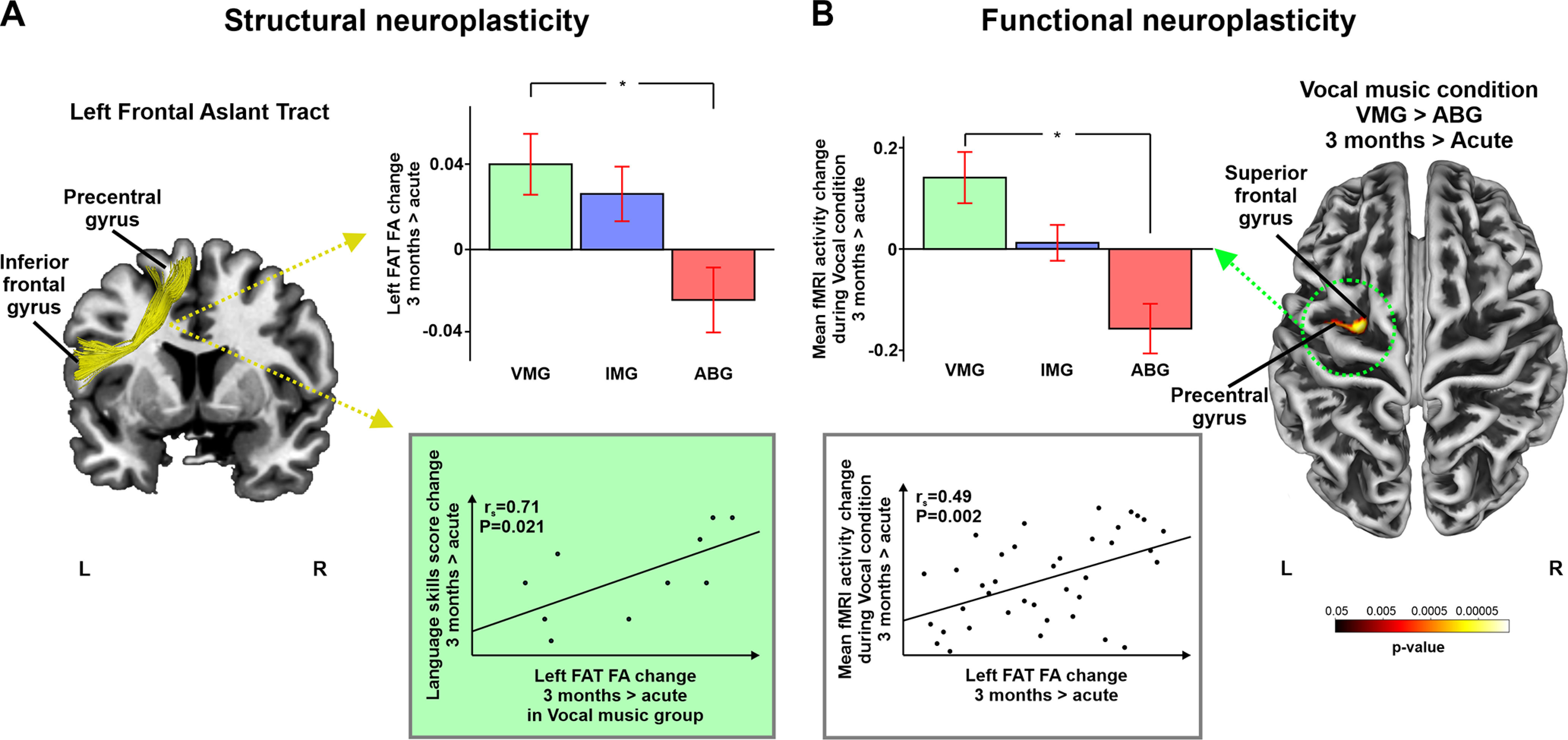
Structural and functional neuroplasticity changes (3 month > Acute). ***A***, Significant tractography results showing increased FA in the left FAT for VMG > ABG (3 month > Acute). Correlations to change in language skills score within the Vocal music group are shown in the scatter plot. ***B***, Significant fMRI-task results showing increased activity between VMG and ABG (3 month > Acute) during vocal music condition. Correlations to increased FA of the left FAT are shown in the scatter plot. Data reported in the histograms are the mean ± SEM. *p < 0.017. L, left; R, right.

Separate univariate ANOVAs for each tract revealed a significant effect on the FA values of the left FAT, indicating that the longitudinal change (3 month > Acute) in the microstructural properties of this tract differed between the groups (*F*_(2,32)_ = 4.819, *p* = 0.015; partial η^2^ = 0.231). *Post hoc t* tests corrected for multiple comparisons (Bonferroni) revealed that the VMG showed a significantly greater increase in FA values over time than the ABG (*p* = 0.017; Fig. 1*A*), whereas there were no significant differences between the IMG and ABG or VMG and IMG. Importantly, the FA change in the left FAT correlated with improved language skills (*r*_s_ = 0.71, *p* = 0.021) within the VMG. There were no significant interactions for the other tracts (AF, IFOF).

### Functional neuroplasticity

Similarly, there was a significant Group × Time interaction for the Vocal > Rest condition revealing that the VMG showed greater longitudinal (3 month > Acute) activation increase than the ABG in a specific left frontal cluster (*p* = 0.016; T = 4.63; size = 216 voxels) located in the superior/middle frontal gyrus and the precentral gyrus (Fig. 1*B*). The increased activity in the significant cluster correlated with the increased FA in the left FAT *r*_s_ = 0.49, *p* = 0.002) across the whole sample. Correlation between the increased activity and improved language skills was nonsignificant. No other significant interactions were detected for the other task conditions (Instrumental > Rest, Speech > Rest).

## Discussion

This study set out to determine the poststroke vocal music listening induced functional and structural neuroplasticity changes in the language network possibly supporting the improved language skills. Our two main findings were that, compared with listening to audiobooks (1) daily poststroke vocal music listening enhanced left FAT structural connectivity, which was linked to better recovery of language skills; and (2) vocal music listening led to increased stimulus-specific functional changes in the superior frontal termination areas of the left FAT that were linked to improved structural connectivity in the left FAT. The present study not only extends previous results on the rehabilitative effects of music listening after stroke (Särkämö et al., 2008, 2014; Baylan et al., 2020; Sihvonen et al., 2020), but also reveals novel information about the neural mechanisms (i.e., functional and structural reorganization of key regions within the language network) that support language recovery in stroke via vocal music listening. This evidence is important in evaluating treatment mediators of music-based rehabilitation strategies (Sihvonen et al., 2017c), and in improving our understanding of aphasia rehabilitation.

Connecting the inferior frontal gyrus with dorsomedial frontal areas and anterior cingulate cortex, the left FAT has recently been recognized as an important tract for speech production (Catani et al., 2012; Thiebaut de Schotten et al., 2012; Dick et al., 2014, 2019; Sierpowska et al., 2015). Damage to the FAT underlies disease-related speech impairments in patients with poststroke aphasia (Basilakos et al., 2014; Halai et al., 2017; Alyahya et al., 2020) and primary progressive aphasia (Catani et al., 2013; Mandelli et al., 2014), as well as in patients with resected frontal gliomas (Kinoshita et al., 2015). Moreover, neuroplasticity changes in the inferior frontal and dorsomedial termination points of the left FAT have been shown to underpin better aphasia outcomes both after targeted rehabilitation and also in patients showing spontaneous recovery (Saur et al., 2006; Schevenels et al., 2020). En masse, while studies evaluating direct aphasia treatment-related structural changes in the left FAT are lacking, its role in regaining language functions after poststroke aphasia as well as a potential target for aphasia treatments stands to reason. The present study suggests that this avenue for poststroke language recovery could be targeted by listening to vocal music.

The sensory and motor environment during the acute stroke stage has a crucial role in the recovery. However, in clinical practice, patients often receive rehabilitation in suboptimal intensity, frequency, and timing (Murphy and Corbett, 2009; Foley et al., 2012), and remain largely inactive and unstimulated during the critical acute stage (Bernhardt et al., 2004; De Wit et al., 2005). In other words, the prerequisites for poststroke rehabilitation exploiting activity-dependent neural plasticity are often not met (Cramer et al., 2011). Music listening could respond to these unmet needs of recovering stroke patients. First, music listening serves as a multimodal stimulus, akin to “enriched environment” where neural stimulation is achieved by increasing stimuli from the physical and social surroundings during the rehabilitation (Nithianantharajah and Hannan, 2006). Studies on healthy subjects have revealed that mere music listening induces a widespread activation pattern in the brain (Schmithorst, 2005; Samson et al., 2011; Alluri et al., 2012; Zatorre and Salimpoor, 2013; Koelsch, 2014). In acute stroke patients, music listening activates a similar network of brain regions (Sihvonen et al., 2017d). This increased neural stimulation supports neural plasticity in the recovering brain by increasing, for example, dendritic spine density and neurotrophic factor levels (Nithianantharajah and Hannan, 2006).

Second, language and music processing have been shown to be supported by common neural networks (Maess et al., 2001; Koelsch et al., 2002; Callan et al., 2006; Schön et al., 2010; Kunert et al., 2015); that is, language network engagement can be modulated by music. This modulatory effect can be enhanced by listening to music with sung lyrics (i.e., vocals), which binds linguistic and musical information into a unified representation: vocal music engages bilateral frontotemporal areas more extensively than speech (Callan et al., 2006; Schön et al., 2010) or music without vocals (i.e., instrumental music; Brattico et al., 2011; Alluri et al., 2013), even in patients with acute stroke (Sihvonen et al., 2017d). Importantly, vocal music engages the left inferior and dorsomedial frontal termination areas of the left FAT, which have been implicated in the auditory–motor processing of music (Zatorre et al., 2007) and singing (Callan et al., 2006). Crucially, the left dorsomedial frontal areas (superior frontal gyrus and anterior cingulate) showed increased gray matter volume after the music listening intervention in our previous stroke study (Särkämö et al., 2014), and the anterior cingulate is also the frontal hub of the default mode network where we previously reported enhanced functional connectivity induced by the vocal music listening (Sihvonen et al., 2020). The present results elaborate the activity-dependent neuroplasticity effects of poststroke vocal music listening by elucidating its effects on modulating communal neural structures for speech and music, underpinning language recovery after stroke.

Regarding the real-world shortcomings in rehabilitation intensity, frequency, and timing, poststroke vocal music listening could be implemented with minimal professional input early in the rehabilitation process (i.e., acute stage). One hour daily patient-led music listening has been shown to be enough in terms of intensity to produce behavioral (Särkämö et al., 2008; Sihvonen et al., 2020) and neuroplasticity (Särkämö et al., 2014; Sihvonen et al., 2020) gains, and, if implemented, could increase the received daily rehabilitation (De Wit et al., 2005) and possibly provide better aphasia outcomes (Bhogal et al., 2003). In turn, this could provide better long-term outcomes by increasing mood and ameliorating social isolation (Doogan et al., 2018).

The present study has some potential limitations. While the randomization to groups was stratified for lesion laterality, the groups showed significant differences for the prevalence of rhythm amusia, the inability to perceive musical rhythm. However, the number of patients with rhythm amusia in each group was largely similar (ranging from 8 to 11). Moreover, the prevalence of amusia and aphasia in the current sample was similar to those in previous studies (Sihvonen et al., 2019), making it representative of the real-world population. Most importantly, despite these group differences, vocal music listening was still effective. To fill the inclusion criteria, patients were required that have some degree of verbal communication and speech comprehension. This means that patients with global or severe aphasia were not able to participate, and aphasic patients in the current study have mild to moderate aphasia. This impedes us from making any conclusion on the effects of vocal music listening in severe forms of poststroke language impairments, and in the future, these effects should be studied. This very limitation can also contribute to the observed pattern of results: in different forms and severities of aphasia, improved outcomes can be underpinned by structural neuroplasticity changes in white matter pathways other than the left FAT (Alyahya et al., 2020; Hula et al., 2020; Gajardo-Vidal et al., 2021). How vocal music listening can target those and possibly mediate recovery is as yet unknown. Moreover, the current sample size prevented us from performing separate analyses for aphasic and nonaphasic patients.

Future research on the effects and the most active ingredients of music listening in poststroke rehabilitation is still needed. One crucial aspect of music is its capacity to evoke and regulate emotions, provide joy and comfort, and relieve stress (Saarikallio, 2011). Emotionally engaging music activates multiple brain circuits related to dopaminergic signaling reward and emotion, the engagement of which has been shown to be directly proportional to the intensity of the experience (Blood and Zatorre, 2001; Salimpoor et al., 2011; Ferreri et al., 2019). The engagement of crucial brain regions related to dopamine, motivation, and pleasure could partly explain the cognitive–emotional gains induced by music listening in neurologic rehabilitation (Sihvonen et al., 2017c). Importantly, the music in the study was self-selected by the patients to maximize personal relevance and emotional arousal, and this could have further enhanced the benefits of poststroke music listening. Future studies should include online (i.e., while the patients listen to music as part of their daily therapy) subjective (e.g., self-reports of pleasure) and objective (e.g., physiological responses via wearable technology that does not hinder the musical experience; Pelofi et al., 2021) measures evaluating the emotional arousal and pleasure of the music material. Furthermore, vocal music listening could be used as an adjuvant therapy in connection with traditional speech therapy to provide neural stimulation and fertile ground for recovery (Nithianantharajah and Hannan, 2006). As a receptive form of music-based rehabilitation, music listening could also support the effects of melodic intonation therapy (Albert et al., 1973), an active, singing-based treatment, the goal of which is to restore propositional speech, that has been related to neuroplasticity effects in the right AF (Schlaug et al., 2009). Furthermore, it would be extremely interesting to investigate whether the beneficial effects of vocal music listening are accessible in patients with different lesion locations affecting the language system (i.e., temporoparietal and frontal strokes; Stockert et al., 2020) and in patients with different aphasia severities and subtypes. Future studies using larger samples should assess the effect that music listening has on more specific language- and auditory-related functions and how this interacts with hemispheric damage. This is of particular importance for language-related functions thought to be supported by bilateral regions in the temporal cortex (Hickok and Poeppel, 2007).

In conclusion, the present results suggest that the positive effects of music listening on stroke recovery are underpinned by structural and functional reorganization of the left hemisphere language network for vocal music. Clinically, the results provide further evidence that vocal music listening is a feasible tool to stimulate the language network and promote language recovery after stroke.

## Synthesis

Reviewing Editor: Sunghee Cho, Burke Neurological Institute/Feil Family Brain and Mind Research Institute, Weill Cornell Medicine Decisions are customarily a result of the Reviewing Editor and the peer reviewers coming together and discussing their recommendations until a consensus is reached. When revisions are invited, a fact-based synthesis statement explaining their decision and outlining what is needed to prepare a revision will be listed below. The following reviewer(s) agreed to reveal their identity: Susan Wortman-Jutt. Note: If this manuscript was transferred from JNeurosci and a decision was made to accept the manuscript without peer review, a brief statement to this effect will instead be what is listed below.

The presented clinical study, describing potential benefit of vocal music listening on post-stroke language therapy, were evaluated by two reviewers with expertise in the field. While both reviewers considered that the manuscript that addresses a potential adjunctive rehabilitation strategy of vocal music listening on the early stage of stroke patients is a timely topic and bears important consequences, they also raised serious concerns. These include the lack of clarity in controlling important variables, the impact of hemispheric differences, and the rationale for the combining three test scores as opposed to present breakdown for each test. It was also noted that missing analyses of an important structure for vocal behavior and insufficient information on the nature of injury. More detailed and specific comments from the reviewers are appended.

Reviewer 1

The authors of the present study propose that listening to vocal music as an adjunct to standard of care post-stroke therapy may provide benefits to stroke patients in the first 3 months of recovery, particularly those with aphasia. This is an important and timely topic.

Imaging data from the authors previous (combined) 2020 study is compared with behavioral data in this manuscript. Findings include microstructural enhancement of the left frontal aslant track when listening to vocal music which the authors correlate with improvement in raw scores of 3 brief, combined language-based subtests. The specifics of what language domains improved most are not yet provided and there are some issues with the study design that may benefit from further explanation. A full language evaluation using a complete standardized assessment is not provided and an explanation of this omission would be helpful. For example, were the authors expecting improvement in "following directions" (as measured by the shortened Token Test), pictured-object naming (as measured by a shortened version of the Boston Naming Test) and word finding (as measured by a fluency task) specifically? And if so, why were those specific areas expected to improve? If not, why was a more thorough and complete language evaluation not conducted?

The authors begin by stating in the Abstract that, "listening to vocal music has been recently shown to improve language recovery in stroke." In their previous (2020) paper from which this manuscript derives, the authors looked at the results of 2 combined studies and found improvements in verbal memory (the primary outcome measure) following 1-hour of passive daily vocal music listening versus listening to audiobooks or instrumental music. In the former paper the authors also found improvement in language scores on 2 abbreviated language subtests (a shortened version of the Token Test and the short version of the Boston Naming Test), and improvements on a verbal fluency task. In the original paper, the authors combine the raw scores from these 3 separate and unrelated subtests into one language score, which, when presented in combination, is neither validated nor standardized. The combination of raw scores from the 3 unrelated subtests into one so-called language score has the potential to be misleading. Table 1 provides mean baseline data on each of the individual language subtests, but it is unclear how each language subtest improved individually over time. In a future version of the current manuscript, it might add merit to provide a breakdown of language scores pre- and post-treatment on all 3 language subtests.

Of note, the authors report that subjects received standard of care therapy in all disciplines throughout the study. They do not report, however, on the timing of the listening relative to participation in therapy. Was it random? Was there a prescribed time set aside for listening relative to when they would receive physical, occupational or speech therapy? If this was not determined or left up to the participant, why was this so? Did the authors not feel there would be a relationship between the timing of their intervention relative to the other interventions the participants were receiving? It would be interesting to understand the rationale behind leaving the timing of listening up to the participants, versus having a prescribed listening period in direct relationship to their other therapies. Having a specified, or even supervised listening period may have helped to ameliorate concerns regarding "cross-listening," which the authors acknowledge (in the original paper) took place, in which the vocal music, instrumental and audiobook groups listened to other types of entertainment on separate devices during the treatment period. Itߣs unclear why a more supervised, structured form of listening given before, say, speech therapy, or before beginning all therapy for the day, wasnߣt prescribed for each group. With respect to the standard of care therapies received during the listening treatment period, it would also be interesting to note whether improvements in language were found by the speech-language therapists during the course of participantsߣ care. Are the findings of the study team consistent with improvements noted by the treating therapists?

Hickok and Poeppel (2007) and later Fridriksson et al. (2016; 2018) have suggested that the right hemisphere is more involved in the auditory comprehension of novel information, such as unfamiliar words. It has been hypothesized by Hickock and Poeppel (2007) that this explains why many patients with fluent aphasia perform well on tests of sound discrimination in spite of their auditory comprehension deficits; that is, there is bilateral representation of this phonological processing skill. This may be relevant to the findings in the present study since the audiobooks contained the rapid presentation of unfamiliar information, whereas the vocal music contained lyrics that may have been overlearned or familiar. Could the automaticity, repetitiveness or over-learned nature (e.g., reduced demand) of the vocal music lyrics have played a role in the outcome? It would be interesting to hear the authorsߣ views; but again, a more complete understanding of what specific language domains were positively impacted by vocal music listening, and whether some domains were impacted more than others, might be helpful.

Finally, one issue confused me with respect to Table 1: Baseline mean Naming scores (BNT) are listed as 18.3, 17.5 and 17.4 respectively; however, the short version of the BNT is standardized as a 15-item test. Was there an alternate number of items used in this "shortened" version of the BNT?

Reviewer 2

This is an innovative intervention and measure in a group of 38 stroke patients with important consequences for post stroke care. Generally, the results are very interesting, however, I would like to have some more information and clarification

1.) sample lacks an empty control/emotional arousal which could account for the differences in outcome

2.) the subgroup are heterogeneous with respect to stroke nature (hemorrhagic vs. ischemic?)

3.) the analysis did not include the most important tract for vocal behavior, the arcuate fasciculus

4.) too little information about the size and nature of the left hemisphere lesion

5.) no discussions of earlier work on melodic intonation therapy by Gottfried Schlaug

6.) What were the DTI results for the FA? This is the fibre bundle which was most commonly investigated - maybe there was no effect, this is interesting (see work on melodic intonation therapy by Schlaug)

7.) Did the subjects rate their pleasure with listening and was this the same (emotional arousal in the three groups?)

8.) is the fact, that in the VMG only 2 patients suffered from hemorrhagic stroke, in the two others however 6 and 4, and therefore a much higher percentage has any impact on the results. I would like to know more about the size of the lesion, the Barthel index etc.etc.

9.) Discussion: Any idea, why MIT is the painful and with so little effect in most patients?
